# The relationship between fatigue, pruritus, and thirst distress with quality of life among patients receiving hemodialysis: a mediator model to test concept of treatment adherence

**DOI:** 10.1038/s41598-024-60679-2

**Published:** 2024-05-01

**Authors:** Hamid Sharif-Nia, João Marôco, Erika Sivarajan Froelicher, Saeed Barzegari, Niloofar Sadeghi, Reza Fatehi

**Affiliations:** 1 Psychosomatic Research Center, Mazandaran University of Medical Sciences, Sari, Iran; 2 Department of Nursing, Amol School of Nursing and Midwifery, Mazandaran University of Medical Sciences, Sari, Iran; 3 William James Centre for Research ISPA–Instituto Universitário, Lisbon, Portugal; 4 Department of Physiological Nursing, School of Nursing, University of California San Francisco, San Francisco, CA USA; 5 Department of Epidemiology & Biostatistics, School of Medicine, University of California San Francisco, San Francisco, CA USA; 6 Department of Paramedicine, Amol School of Paramedical Sciences, Mazandaran University of Medical Sciences, Sari, Iran; 7 Student Research Committee, Mazandaran University of Medical Sciences, Sari, Iran

**Keywords:** Fatigue, Hemodialysis, Pruritus, Quality of life, Thirst distress, Treatment adherence, Diseases, Health care, Nephrology, Signs and symptoms

## Abstract

Hemodialysis is a conservative treatment for end-stage renal disease. It has various complications which negatively affect quality of life (QOL). This study aimed to examine the relationship between fatigue, pruritus, and thirst distress (TD) with QOL of patients receiving hemodialysis, while also considering the mediating role of treatment adherence (TA). This cross-sectional study was carried out in 2023 on 411 patients receiving hemodialysis. Participants were consecutively recruited from several dialysis centers in Iran. Data were collected using a demographic information form, the Fatigue Assessment Scale, the Thirst Distress Scale, the Pruritus Severity Scale, the 12-Item Short Form Health Survey, and the modified version of the Greek Simplified Medication Adherence Questionnaire for Hemodialysis Patients. Covariance-based structural equation modeling was used for data analysis. The structural model and hypothesis testing results showed that all hypotheses were supported in this study. QOL had a significant inverse association with fatigue, pruritus, and TD and a significant positive association with TA. TA partially mediated the association of QOL with fatigue, pruritus, and TD, denoting that it helped counteract the negative association of these complications on QOL. This model explained 68.5% of the total variance of QOL. Fatigue, pruritus, and TD have a negative association with QOL among patients receiving hemodialysis, while TA reduces these negative associations. Therefore, TA is greatly important to manage the associations of these complications and improve patient outcomes. Healthcare providers need to assign high priority to TA improvement among these patients to reduce their fatigue, pruritus, and TD and improve their QOL. Further studies are necessary to determine the most effective strategies for improving TA and reducing the burden of complications in this patient population.

## Introduction

Chronic kidney disease (CKD) is one of the most prevalent chronic illnesses throughout the world. It involves structural injuries to the kidney and a glomerular filtration rate (GFR) below 60 mL/min/1.73 m^2^ persisting for at least 3 months^1^. The global prevalence of CKD is as high as 10% or 800 million people^2^. Its prevalence is 8–16% in the world^3^ and almost 20% in Iran^4^. Predictions show that CKD will be the fifth leading cause of death in the world by 2040^5^.

Currently, hemodialysis is the most common treatment option for CKD before kidney transplantation^6^. Hemodialysis is a procedure that filters a patient's blood through a machine, removing waste substances and excess water by passing them across a semi-permeable membrane^7^. Almost 69% of all patients with CKD^8^ and 49% of them in Iran receive hemodialysis^9^. A study reported that in 2016, around 30,000 patients in Iran received hemodialysis^6^.

CKD and hemodialysis cause different complications^10^, including fatigue. By definition, fatigue is a feeling of a lack of energy that interferes with the performance of daily activities^11^. Its prevalence among patients receiving hemodialysis is 60–80%^12^. The contributing factors to fatigue among these patients include anemia, uremia, inappropriate diet, hemodialysis inefficiency, sedentary lifestyle, sleep pattern disorders, fluid restriction, dehydration, and other comorbid conditions^13,14^. Fatigue can lead to mental and physical weakness, non-adherence to medications, absence from hemodialysis sessions, depression, and heavy costs for patients and societies^15,16^. Fatigue-induced depression and altered immunity can also lead to skin inflammation, skin infections, and pruritus^17,18^.

Pruritus is very common among patients who receive hemodialysis. Uremic pruritus is attributed to CKD and end-stage renal disease in the absence of primary dermatologic findings and other pruritus-inducing disorders such as eczema^19^. With a prevalence of 20%–50%, pruritus is one of the most common contributing factors to discomfort in CKD^20^. The major causes of pruritus in CKD are cytokines, hyperparathyroidism, hyperkalemia, hyperphosphatemia, uremia, anemia^21^, and waste product accumulation in the skin^22^. Pruritus can lead to erosion at the vascular access site and loss of the hemodialysis session^19^.

Thirst distress (TD) is another very common complication of CKD and hemodialysis with a prevalence of 67–97%^23^. TD is a subjective perception defined as a sense of mouth dryness with a desire for fluid intake^24^. Patients with CKD suffer from impaired urine production and hence the intake of foodstuffs and fluids can lead to fluid overload and hypoosmolality. Hence, increased desire for salt intake which is associated with TD. TD can also lead to dryness in the mouth, reduced energy, and fatigue^25,26^. These complications may interact with each other, leading to a harmful cycle of negative physical and mental health outcomes.

Fatigue^13^, pruritus^21^, and TD^25^ among patients receiving hemodialysis can negatively affect their QOL. QOL among these patients refers to general well-being, satisfaction, and ability to perform daily activities while receiving hemodialysis, and includes physical health, psychoemotional well-being, social functioning, and general satisfaction with life^27^. Fatigue can reduce patients’ QOL by reducing their motivation for treatment continuation, impairing their social relationships, and reducing their sleep quality^11^. Pruritus also reduces their QOL by disturbing the patient's social balance, causing them fatigue, increasing their anxiety, and impairing their daily activities^19^. TD also negatively affects their QOL because the necessity of fluid restriction causing them to feel thirsty, which in turn leads to negative emotions such as guilt, anxiety, and discomfort^25^. A systematic review of 45 studies with a total population of 17,000 patients with CKD reported that they had poor QOL^10^. Several other studies also reported low to moderate QOL among these patients^28–32^.

Treatment adherence (TA) is one of the factors with a potentially positive impact on CKD and hemodialysis complications, patient’ QOL^33^, and hemodialysis outcomes^34^. It consists of adherence to dietary regimen, medications, fluid restriction, and regular attendance at hemodialysis sessions^34^. By definition, TA refers to patients’ active engagement in a course of acceptable health-related behaviors which lead to positive treatment outcomes^35^. As hemodialysis is not a perfect substitute for kidneys, TA is of great importance to maintaining the patient’s health^36^, so hemodialysis has limited efficiency in the absence of TA^37^. Patients receiving hemodialysis need to restrict phosphorus intake while they need to receive adequate protein to prevent malnutrition^38^. Moreover, their adherence to salt restriction can reduce their TD^39^. Close adherence to pharmacological and non-pharmacological therapies such as nutrition therapy, sleep improvement modalities, stress management, physical exercise, and yoga can reduce fatigue among these patients^40^. Moreover, adherence to treatments for anemia can increase hemoglobin levels and oxygen transport and thereby, reduce fatigue^41^. Nonetheless, a study reported that poor TA among patients receiving hemodialysis is as high as 50%^42^. Another study showed that 86% of patients receiving hemodialysis had poor adherence to dietary regimens and 86% of them did not adhere to some dietary restrictions^43^. Similarly, a study revealed that 33% of these patients had poor adherence to fluid restriction^44^. Another study also found that one-fourth of patients with end-stage renal disease had a history of absence from hemodialysis sessions during the past month^45^. Generally speaking, estimations show that non-adherence to medication regimens among these patients varies from 3 to 80%^46^. Such poor TA can increase the complications of hemodialysis^42,47^. Moreover, the absence of hemodialysis sessions increases waste products in the body^48^ and increases the mortality rate^42^. Contributing factors to poor TA include the large number and unpleasant taste of some medications, the complexity of treatments^49^, and the side effects of medications^50,51^.

### Literature review

Previous studies on patients receiving hemodialysis reported the inverse relationship of their QOL with their fatigue^52,53^, pruritus^18,54,55^, and TD^25,49,56^. The QOL of these patients is also affected by factors such as the long-term course of their treatment, complex treatment regimens, dermatologic manifestations, and lifestyle changes^57^. These findings denote that the effective management of these problems may improve patients’ QOL. Respecting TA, different studies found a positive correlation with the QOL of patients receiving hemodialysis^58–61^. However, some studies reported no significant relationship between TA and QOL^62^. Moreover, there is limited data about the combined association of fatigue, pruritus, TD, and TA with QOL. Therefore, the current study was conducted in order to examine the inter-relationships of fatigue, pruritus, and TD with QOL and the potential mediating role of TA among patients receiving hemodialysis.

### Conceptual framework of the study

The conceptual framework of this study was the symptom management theory (SMT). This theory emphasizes the importance of symptom management to improve QOL^63^. The four components of this theory are symptom experience, symptom management strategies, outcomes, and influential factors on symptom management. This theory focuses on the perception and management of the symptoms that patients experience with chronic illnesses. It focuses on the dynamic and multidimensional characteristics of the symptoms while considering the complex interaction of biological, psychological, and social factors that influence symptom experience. It also provides a comprehensive approach to evaluate, measure, and manage symptoms to improve QOL among different patient populations. This theory considers symptom management as a complex and multidisciplinary phenomenon that needs the collaboration and interaction of healthcare providers, patients, and families^64,65^. SMT is a critical approach that aims to address and alleviate symptoms experienced by patients to enhance their QOL^66^. Specifically in the context of hemodialysis, this theory plays a crucial role in controlling common side effects such as fatigue, pruritus, and TD. By understanding the interconnected nature of symptoms and their impact on patient's well-being, healthcare providers can develop personalized interventions to effectively manage these symptoms. The theory underscores the significance of tailored and precise symptom management strategies to enhance patient outcomes^67–69^.

By utilizing the SMT, healthcare providers can implement comprehensive symptom management programs that address these side effects holistically. Through a systematic approach that takes into account the synergy between symptoms, providers can effectively classify and manage symptom clusters. This shift in focus from individual symptoms to interconnected symptom clusters allows for a more targeted and comprehensive management strategy^69,70^.

By understanding the relationships between symptoms such as fatigue, pruritus, and TD, healthcare providers can develop interventions that not only alleviate these side effects but also enhance treatment adherence. TA may serve as a mediating factor that helps mitigate the negative effects of symptoms on QOL. Therefore, by integrating the principles of SMT into practice, healthcare providers can optimize patient care by effectively controlling common side effects associated with hemodialysis, ultimately leading to improved patient outcomes^68,71^.

### Research problems

Based on the literature and proposed conceptual model, the following hypotheses are raised:

1. Fatigue reduces the QOL of patients undergoing hemodialysis.
2. Pruritus reduces the QOL of patients undergoing hemodialysis.
3. TD reduces the QOL of patients undergoing hemodialysis.
4. TA increases the QOL of patients undergoing hemodialysis.
5. The mediating role of TA may explain the relationship between fatigue and QOL of patients undergoing hemodialysis.
6. The mediating role of TA may explain the relationship between pruritus and QOL of patients undergoing hemodialysis.
7. The mediating role of TA may explain the relationship between TD and QOL of patients undergoing hemodialysis.

Responding to these problems can help clarify if addressing adherence is crucial for improving patients' well-being beyond managing only the complications.

## Methods

### Design and participants

This cross-sectional study was carried out using structural equation modeling between September to October 2023.

The population of the study consisted of all patients with CKD who received hemodialysis. Participants were consecutively selected from four hospitals in Amol, Iran. Inclusion criteria were to be above eighteen years old and to have received hemodialysis for at least one year before the study. Exclusion criteria were peritoneal dialysis, kidney transplantation, emergency hemodialysis, and acute renal failure during the study. The sample size was calculated for structural equation modeling^72^ with a moderate effect size of 0.25^73^, a power of 0.80, a confidence level of 0.95, five latent factors, and 48 items of the data collection instruments. Calculations showed that 229 participants were necessary. Nonetheless, considering an attrition rate of at least 10% due to missing data, the sample size was increased to 252 and finally, 411 patients were recruited to the study.

### Data collection instruments

The instruments for data collection were as follows.

#### Demographic information form

This data collection form included items on age, gender, employment status, level of education, marital status, social support, duration of hemodialysis sessions, time on hemodialysis, and affliction by comorbid chronic illnesses.

#### Fatigue Assessment Scale (FAS)

Michielsen et al. developed this 10-item scale in 2003. It has five items on physical fatigue and five items on mental fatigue. The items are scored on a five-point Likert scale from 1 (“Never”) to 5 (“Always”). Items 4 and 10 are reversely scored. The possible total score of the scale ranges from 10 to 50, with scores less than 22 showing no fatigue and scores 22 and more showing fatigue. In other words, higher scores show greater fatigue^74^. The reliability and validity of this scale for patients with sarcoidosis in Iran have been confirmed, with a Cronbach's alpha coefficient of 0.927^75^. The reliability of this scale in the present study was confirmed with a Cronbach’s alpha of 0.807, a McDonald’s omega of 0.816, and an average inter-item correlation coefficient (AIC) of 0.293.

#### Thirst Distress Scale (TDS)

Welch and Molzahn developed this scale in 2002 for patients receiving hemodialysis. It has six items scored on a five-point Likert scale from 5 (“Strongly agree”) to 1 (“Strongly disagree”). Its possible total score is 6–30 and higher scores show greater TD^76^. The Cronbach’s alpha, McDonald’s omega, and an AIC of this scale in the present study were respectively 0.891, 0.967, and 0.574, which confirmed its acceptable reliability.

#### The 12-Item Pruritus Severity Scale (12-PSS)

This scale was developed in 2017 by Reich et al. for patients with chronic pruritus. It has twelve items on the five main dimensions of pruritus, namely pruritus intensity, pruritus frequency, pruritus duration, pruritus influence on daily activities and mood, and scratching as a response to pruritus. Items are scored differently (including yes/no items and items with different Likert scales) and the possible total score of the scale ranges from 3 (minimum pruritus) to 22 (maximum pruritus). The total score is categorized as follows: scores 3–6: mild pruritus; scores 7–11: moderate pruritus; and scores 12–22: severe pruritus^77^. The reliability of this scale for hemodialysis patients in Iran has been examined and confirmed, with a Cronbach's alpha of 0.890^78^. The reliability of the scale was confirmed in the present study with a Cronbach’s alpha of 0.850, a McDonald’s omega of 0.862, and an AIC of 0.341.

#### The 12-ItemShort Form Health Survey (SF-12)

Ware et al. developed this twelve-item scale for QOL assessment based on the original 36-item Health Survey. It predicts 90% of the variance of the 36-item Health Survey^79^. It has two main dimensions, namely physical health and mental health, with the eight subscales of general health perception, physical functioning, role limitations due to physical health, bodily pain, role limitations due to emotional problems, social functioning, vitality, and mental health. Two items are Yes/No questions and ten items are scored using various Likert scales. Items 1, 8, 10, and 11 are reversely scored. The possible range of the total score is 12–48, with higher scores standing for better QOL. Scores 12–24, 25–36, and 37–48 show poor, moderate, and good QOL, respectively. The reliability of this survey in Iran for the general population has been thoroughly examined and confirmed. The Cronbach's alpha for the physical and mental components was found to be 0.730 and 0.720, respectively. This indicates a high level of internal consistency and reliability in the survey results^80^. The Cronbach’s alpha, McDonald’s omega, and an AIC of the scale in the present study were 0.803, 0.836, and 0.312, respectively.

#### A modified version of the Greek simplified medication adherence questionnaire for hemodialysis patients (GR-SMAQ-HD)

Alikari et al. developed this eight-item questionnaire in 2017 for patients receiving hemodialysis. Three items are Yes/No questions and five items are scored on a five-point Likert scale. Items are on the different aspects of TA, namely medication adherence, attendance at hemodialysis sessions, and fluid/diet restrictions. Items are scored either zero or 1 and hence, the possible range of the total score of the questionnaire is 0–8, where higher scores show greater TA^81^. In the present study, the Cronbach’s alpha, McDonald’s omega, and an AIC of items 1–4 were 0.734, 0.744, and 0.389, the Cronbach’s alpha and an AIC of items 5 and 6 were 0.894 and 0.810, and the Cronbach’s alpha and an AIC of items 7 and 8 were 0.582 and 0.412, respectively.

### Data analysis

The Kaiser–Meyer–Olkin and Bartlett’s tests were used to determine sampling adequacy and model appropriateness in factor analysis. Kaiser–Meyer–Olkin test values of more than 0.7 show model appropriateness^82^. The Mahalanobis distance was also used to find multivariate outliers^83^. Univariate normality was tested via skewness (± 3) and kurtosis (± 7) measures and multivariate normality was tested via the Mardia’s coefficient (< 8)^83^. Finally, structural equation modeling was performed to assess the mediating role of TA in the relationship of fatigue, pruritus, and TD with QOL. Bootstrapping with 2000 repetitions was also employed for hypothesis testing in structural modeling^84^. Model fit indices were root mean square error of approximation (RMSEA; < 0.08), Standardized Root Mean Square Residual (SRMR; < 0.10), comparative fit index (CFI; > 0.90), incremental fit index (IFI; > 0.90), and Tucker-Lewis index (TLI; > 0.90)^83^. The Internal consistency was tested using Cronbach’s alpha, McDonald’s omega, AIC, and composite reliability (CR), where Cronbach’s alpha, McDonald’s omega, and CR values of more than 0.7, and AIC values of 0.2–0.4 were interpreted as acceptably reliability^83^. Statistical analyses were conducted using the SPSS (v. 26.0), AMOS (v. 27.0), and RStudio Integrated Development Environment (v. 4.1.0) software. All statistical hypotheses were two-tailed and the significance levels were set at less than 0.05.

### Ethics

This study obtained approval from the Ethics Committee of Mazandaran University of Medical Sciences, Sari, Iran (code: IR.MAZUMS.REC.1402.344). Data collection took place after explaining the study's purpose to participants, ensuring their voluntary participation and data confidentiality. Written Informed consent was obtained from all subjects and/or their legal guardian(s). Permissions to use the data collection tools were acquired from their developers. All procedures adhered to the appropriate guidelines and regulations.

## Results

None of the participants were excluded from the study and the data of all 411 recruited participants were analyzed. The participants’ mean age was 59.37 (SD =  ± 12.99) years (95% confidence interval: 58.11–60.63) and the percentage of men and women participants was almost equal (50.1% vs. 49.9%). Most participants reported that they had social support (78.10%). Table 1 shows participants’ characteristics.

**Table 1:** Demographic profiles of the participants (n = 411).

Variables	Mean (SD)	
Age	59.37 (± 12.99)	
Duration of hemodialysis (year)	4.90 (± 4.01)	

Variables		n (%)
Gender	Men	206 (50.1)
	Women	205 (49.9)
Marital status	Single	38 (9.2)
	Married	373 (90.8)
Social support	Yes	321 (78.1)
	No	90 (21.9)
Time on hemodialysis	Morning	160 (38.9)
	Evening	140 (34.1)
	Night	111 (27.0)
Level of education	Below diploma	232 (56.4)
	Diploma	62 (15.1)
	University	117 (28.5)
Employment status	Housewife	183 (44.5)
	Manual worker	20 (4.9)
	Employee	25 (6.1)
	Self-employed	93 (22.6)
	Unemployed	34 (8.3)
	Retired	56 (13.6)
Chronic disease	Diabetes mellitus	240 (58.4)
	Heart disease	219 (53.3)
	Respiratory disease	47 (11.4)
	Anemia	282 (68.6)
	Thyroid disease	107 (26.0)
	Blood Pressure	317 (77.1)

**Figure 1:**
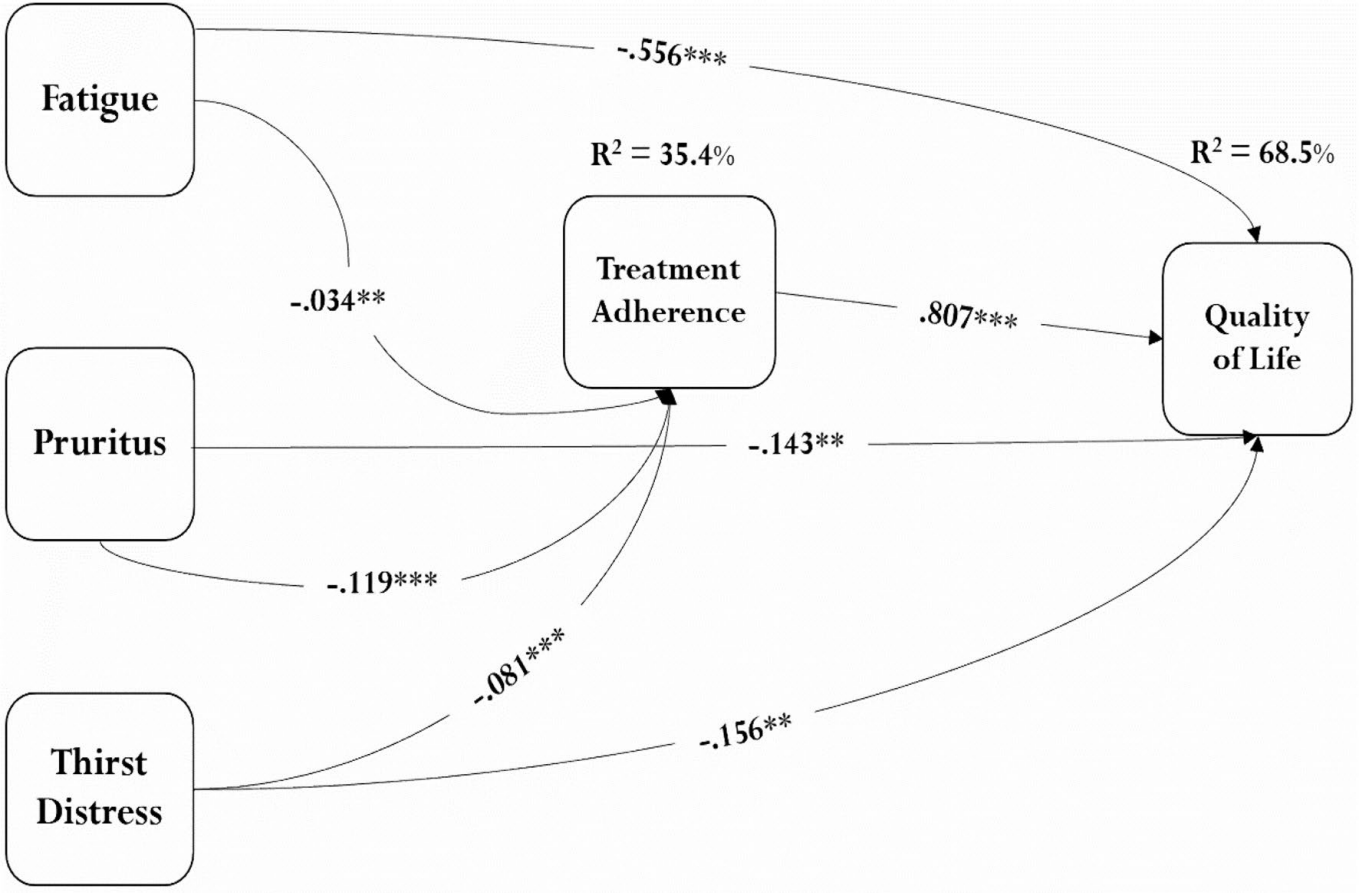
The results of the mediation model assessment; ***p < 0.001, **p < 0.01, *p < 0.05.

Structural equation modeling with bias-corrected bootstrapping and 2000 repetitions showed that the model fit indices were appropriate (CFI = 0.99, TLI = 0.99, IFI = 0.99, RMSEA = 0.09, and SRMR = 0.11). The mediation model was tested by controlling the effects of gender, age, level of education, marital status, social support, time on hemodialysis and duration of hemodialysis sessions, employment status, and affliction by chronic illnesses. The results of the direct effects showed a significant inverse relationship between fatigue and QOL (b = − 0.584, r = − 0.798, p-value < 0.001), between pruritus and QOL (b = − 0.240, r = − 0.563, p-value < 0.001), and between TD and QOL (b = –0.222, r = 0.615, p-value < 0.001). Moreover, there was a significant positive relationship between TA and QOL (b = 0.807, r = 0.568, p-value < 0.001) (Table 2 and Fig. 1).

**Table 2:** The mediation model assessment.

SEM	b	p-value	95% confidence level		r
			Lower bound	Upper bound	
Direct effects					
Fatigue → quality of life	− 0.584	***	− 0.805	− 0.695	− 0.798
Pruritus → quality of life	− 0.240	***	− 1.149	− 0.862	− 0.563
Thirst distress → quality of life	− 0.222	***	− 1.014	− 0.789	− 0.615
Treatment adherence → quality of life	0.807	***	2.204	2.926	0.568
Fatigue → treatment adherence	− 0.034	0.006	− 0.122	− 0.087	
Pruritus → treatment adherence	− 0.119	***	− 0.232	− 0.166	
Thirst distress → treatment adherence	− 0.081	***	− 0.185	− 0.130	
Indirect effects					
Fatigue → quality of life	− 0.087	***	− 0.705	− 0.585	
Pruritus → quality of life	− 0.305	***	− 0.815	− 0.510	
Thirst distress → quality of life	− 0.208	***	− 0.769	− 0.532	
Full effects					
Fatigue → quality of life	− 0.556	***	− 0.633	− 0.480	
Pruritus → quality of life	− 0.143	0.025	− 0.270	− 0.017	
Thirst distress → quality of life	− 0.156	0.005	− 0.265	− 0.047	

***p < 0.001, **p < 0.01, *p < 0.05, , two-tailed test.

Findings revealed a significant indirect effect of TA in the relationships between fatigue and QOL (b = − 0.087, p-value < 0.001), between pruritus and QOL (b = − 0.305, p-value < 0.001), and between TD and QOL (b = − 0.208, p-value < 0.001) (Table 2 and Fig. 1). Figure 1 shows the full effect and depicts the results of the structural model assessment, in which the mediation model accounted for 35.4% of the variance of TA and 68.5% of the total variance of QOL.

Table 3 displays the reliability of the scales, and questionnaires, along with the factor loading of each item. Instruments showed acceptable reliability. Items 8 of the FAS, 5 of the 12-PSS, 2 and 5 of the TDS, and 3 of the GR-SMAQ-HD for hemodialysis patients were removed.

**Table 3:** The factor loading values and reliability parameters of the study instruments.

Construct	Factor loading	Reliability	CR	AVE
Fatigue				
Item 1: I am bothered by fatigue	0.807	α = 0.807, Ω = 0.816 AIC = 0.293	0.819	0.455
Item 2: I get tired very quickly	0.766			
Item 3: I don't do much during the day	0.240			
Item 4: I have enough energy for everyday life	0.619			
Item 5: Physically, I feel exhausted	0.679			
Item 6: I have problems to start things	0.505			
Item 7: I have problems to think clearly	0.667			
Item 8: I feel no desire to do anything	0.290			
Item 9: Mentally, I feel exhausted	0.629			
Item 10: When I am doing something, I can concentrate quite well	0.458			
Pruritus				
Item 1: How often did you feel pruritus within the last 3 days?	0.738	α = 0.850, Ω = 0.862 AIC = 0.341	0.873	0.804
Item 2: Did pruritus hinder your ability to do simply things, like watching TV, hearing music, etc.?	0.977			
Item 3: Did you feel irritated or nervous because of your itching?	0.923			
Item 4: Did your pruritus cause you depressed?	0.914			
Item 5: Did your pruritus impede your work or learning abilities?	1.003			
Item 6: Did you scratch your skin because of itching?	0.790			
Item 7: Did scratching bring you relief?	0.156			
Item 8: Were you able to refrain from scratching?	0.653			
Item 9: Did you wake up during last night because of pruritus?	0.712			
Item 10: Could you assess the severity of your pruritus within last 3 days?	0.544			
Item 11: Could you indicate pruritus location?	0.559			
Item 12: Are excoriations or other scratch lesions present?	0.299			
Thirst distress				
Item 1: My thirst causes me discomfort	0.967	α = 0.891, Ω = 0.967 AIC = 0.574	0.967	0.877
Item 2: My thirst bothers me a lot	1.004			
Item 3: I am very uncomfortable when I am thirsty	0.994			
Item 4: My mouth feels really dry when I am thirsty	0.989			
Item 5: My saliva is very thick when I am thirsty	1.000			
Item 6: Did you scratch your skin because of itching?	0.593			
Quality of life				
Item 1: In general, would you say your health is:	0.520	α = 0.803, Ω = 0.836 AIC = 0.312	0.837	0.493
Item 2: Moderate Activities, such as moving a table, pushing a vacuum cleaner, bowling, or playing golf:	0.654			
Item 3: Climbing Several flights of stairs:	0.616			
Item 4: Accomplished Less than you would like:	0.623			
Item 5: Were limited in the Kind of work or other activities:	0.510			
Item 6: accomplished Less than you would like:	0.962			
Item 7: Didn’t do work or other activities as Carefully as usual:	0.944			
Item 8: how much did Pain interfere with your normal work (including both work outside the home?	0.504			
Item 9: how much of the time have your physical or emotional problems interfered with your social activities?	0.482			
Item 10: Have you felt calm and peaceful?	0.820			
Item 11: Did you have a lot of energy?	0.900			
Item 12: Have you felt downhearted and blue?	0.632			
Treatment adherence				
Item 1:When you feel bad, have you ever stopped taking your medications?	0.497	α = 0.734, Ω = 0.744 AIC = 0.389	0.846	0.665
Item 2: Have you ever forgotten to take your medications?	0.977			
Item 3: Have you ever forgotten to take your medications on the days between the two dialysis sessions?	1.007			
Item 4: In the last week, how many times have you not taken your medications?	0.666			
Item 5: Last month, how many times was the session shortened on your own initiative?	0.908	α = 0.894 AIC = 0.810		
Item 6: Last month, on average, how many minutes was the session cut off on your own initiative?	0.893			
Item 7: During the past week, how many times did you follow fluid restrictions?	0.519	α = 0.582 AIC = 0.412		
Item 8: During the past week, how many times did you follow dietary recommendations?	0.746			

α: Cronbach’s alpha; Ω: McDonald's omega; AIC: average inter-item correlation; CR: composite reliability; AVE: average variance extracted.

## Discussion

The relationship between fatigue, pruritus, TD, and QOL among patients undergoing hemodialysis can be better understood through SMT, with TA playing a crucial role as a mediating variable. Fatigue, pruritus, and TD are common complications experienced by hemodialysis patients, significantly associated with their QOL. Research findings indicate that higher levels of fatigue, pruritus, and TD are linked to lower QOL scores, highlighting the detrimental impact of these complications on overall well-being. However, the positive correlation between TA and QOL suggests that following treatment plans can help alleviate the negative effects of these complications on QOL. TA acts as a protective factor, reducing the adverse impact of complications and improving patient outcomes.

The finding revealed a significant negative relationship between fatigue and QOL. In line with this finding, patients receiving hemodialysis in a previous study reported impaired QOL due to the loss of energy, reduced cognitive and motor functioning, increased dependence, and reduced self-esteem^13^. Fatigue is a mental state of burnout with reduced motivation, altered social relationships, and reduced sleep quality and thereby, can reduce QOL among patients receiving hemodialysis. Chronic fatigue in hemodialysis patients may be attributed to anxiety, depression, and poor sleep quality. These factors can result in decreased motivation, changes in social interactions, and a diminished overall QOL for individuals undergoing hemodialysis treatment^85^.

A study on patients receiving hemodialysis reported that anxiety and depression had a significant relationship with fatigue^86^. Anxiety and depression are prevalent among hemodialysis patients, significantly impacting their QOL. These conditions are closely linked to fatigue, demonstrating their collective influence on patient well-being. Individuals suffering from anxiety and depression are more prone to experiencing fatigue, highlighting the interconnected nature of these factors and their combined effect on QOL^87^.

Increased levels of the metabolites of tryptophan and the precursors of serotonin and melatonin among patients receiving hemodialysis are associated with depression and fatigue^88^. Moreover, sleep disorders among these patients threaten their general health, mental health, and physical capacity, and cause them fatigue. A study indicated a significant relationship between sleep disorders and fatigue among patients receiving hemodialysis and noted that the effective management of sleep disorders can reduce their fatigue and improve their QOL^89^.

Inadequate sleep quality is prevalent among hemodialysis patients and is associated with feelings of fatigue, anxiety, and depression. Issues such as insomnia and daytime sleepiness are frequently experienced by these individuals, exacerbating their fatigue and depression levels and ultimately affecting their overall QOL. It is crucial to address sleep disturbances, as well as effectively manage fatigue, anxiety, and depression, to enhance the well-being and QOL of hemodialysis patients^90^. Moreover, the elimination of waste products from the body during hemodialysis can also lead to hemodynamic instability, blood pressure fluctuations, electrolyte imbalances, and thereby, fatigue and energy loss^91^ (Hypothesis-1).

We also found a significant inverse relationship between pruritus and QOL. This finding is consistent with the findings of a study that reported that uremic pruritus reduced sleep quantity and quality among patients receiving hemodialysis and thereby, reduced their QOL^54^. Patients with severe pruritus are more likely to stay awake at night, feel sleepy during the day, and have inadequate sleep^92^. A study also showed that dermatologic problems and altered body image due to pruritus and erosion reduced QOL among patients receiving hemodialysis^93^. Moreover, aesthetic problems and pruritus caused these patients occupational dysfunction and social isolation which in turn negatively affected their disease burden, daily life, and QOL^93^ (Hypothesis-2).

Our findings also demonstrated a significant inverse relationship between TD and QOL. A study on patients receiving hemodialysis reported that fluid restriction caused these patients problems such as thirst, guilt, distress, and anxiety, while their constant exposure to thirst caused them fatigue and reduced their QOL^26^. Dryness of the mouth due to fluid restriction has a direct relationship with thirst and increases the risk of weight gain and orodental problems such as bacterial and fungal infections, candidiasis, dental caries, and periodontal diseases^94^. These problems cause difficulty in speaking, chewing, and eating, and thereby, greatly affect oral health and QOL^95^ (Hypothesis-3).

Another finding of the present study was the significant positive relationship between TA with QOL. Similarly, a study showed that low medication adherence had a significant inverse correlation with physical QOL^96^. The close adherence of patients receiving hemodialysis to their treatment regimen significantly improves all dimensions of their QOL and reduces their vulnerability and hemodialysis complications. Moreover, adherence to dietary restrictions, fluid restrictions, and medications significantly reduces symptoms and medication side effects and thereby, improves QOL and hope among patients^43^. Close TA also allows patients receiving hemodialysis to have an active role in their care, improves their sense of control and empowerment, and enhances their mental well-being^97^ (Hypothesis-4).

Findings also indicated that TA indirectly affects QOL through mediating the fatigue-QOL relationship. Because of treatment-induced fatigue, patients receiving hemodialysis feel energy depletion and physical exhaustion and need more energy and time to adhere to their strict treatment regimen^98^. Fatigue can also negatively affect patients’ attendance at hemodialysis sessions, while timely attendance at the sessions can improve hemodialysis efficiency, reduce fatigue, and improve QOL^99^ (Hypothesis-5).

We also found the indirect effect of TA on QOL through mediating the relationship between pruritus and QOL. Pruritus reduces sleep quality, increases fatigue, and thereby, negatively affects the different aspects of TA such as attendance at hemodialysis sessions^92^. Poor adherence to hemodialysis sessions and medications obviously increases the need for re-hospitalization, imposes added costs on patients and healthcare systems, and reduces patients’ physical and mental QOL^100^. Conversely, close TA can reduce pruritus and discomfort and hence, improve QOL, social interactions, and self-confidence, and increase patients’ ability to perform their daily activities^101^. On the other hand, effective management of pruritus enhances patient satisfaction with treatment which in turn improves their TA in a virtuous cycle and ultimately improves QOL and treatment outcomes^102^ (Hypothesis-6).

We also found the significant mediating role of TA in the relationship of TD with QOL. Physiological symptoms such as TD and mouth dryness are one of the major barriers to adherence to fluid restriction^25^. Therefore, improving adherence to fluid restriction can reduce the overconsumption of fluids and weight gain between hemodialysis sessions and thereby, can maintain electrolyte balance and improve treatment outcomes^103^. Besides, adherence to dietary regimens, such as limited salt intake, can reduce TD which is a major contributing factor to fluid overconsumption^39^ (Hypothesis-7).

The findings not only support the SMT but also highlight the crucial relationship between complications such as fatigue, pruritus, and TD, as well as TA, and QOL in hemodialysis patients. Improving TA is identified as a key strategy to reduce the negative impact of complications on QOL. This underscores the importance of comprehensive care approaches that focus on both symptom management and TA to enhance patient outcomes in this population. Healthcare providers should use these study results to improve patient outcomes by focusing on symptoms like fatigue, pruritus, and TD in hemodialysis patients. They can tailor interventions, emphasize TA, educate patients, monitor symptoms, and provide collaborative care to enhance QOL. By addressing these specific complications and promoting patient involvement in their care, healthcare providers can work towards better health outcomes for hemodialysis patients.

## Limitations

Like all studies, this study had some limitations. For example, the study is limited by its cross-sectional design, which does not allow for causal conclusions to be reached. The sample size may be small and not representative of the larger population of patients undergoing hemodialysis. As study data were collected through the self-report method, fatigue might have affected participants’ desire to participate in the study, and the accuracy, and concentration for answering the study instruments. We attempted to manage this limitation by providing participants with clear explanations about the study's aim and methods and providing them with adequate time to provide answers to the instruments. Moreover, some participants could not personally complete the study instruments due to problems such as arteriovenous fistula in the limb or low literacy level. We did our best to manage this limitation by involving their companions in data collection and using the interview method for data collection.

## Conclusion

This study suggests that patients receiving hemodialysis can reduce their fatigue, pruritus, and TD and improve their QOL through close TA. Healthcare providers need to improve their knowledge about influential factors on QOL among these patients and employ appropriate interventions to improve their TA, reduce hemodialysis-related complications, and improve their QOL and clinical conditions. Understanding the mediating role of TA can clarify how these complications are associated with QOL and offer valuable insights for developing targeted interventions, education, and strategies to improve QOL in hemodialysis patients. Additionally, comprehending the relationships of these factors with QOL allows patients to effectively communicate their experiences and seek appropriate support, ultimately leading to improved care and outcomes. It can also aid in developing evidence-based guidelines to manage complications and promoting adherence in hemodialysis patients. The results could change clinical guidelines and policies, through the management of complications for hemodialysis patients. This might include regular screening and incorporating complication management into care plans.

## Recommendations

Further studies are necessary to assess the mediating role of the different dimensions of TA in the relationship of different hemodialysis complications with QOL. Moreover, the structural equation modeling approach used in the present study is recommended to assess the association of TA on patient outcomes among patients with cardiovascular disease, particularly hypertension. Studies on patients receiving hemodialysis are also necessary to assess the relationship of fatigue, pruritus, and TD with age, gender, and comorbid illnesses in order to identify patients who may be more susceptible to complications of hemodialysis. Adequate knowledge about these complications and their contributing factors helps healthcare providers use more effective strategies for fulfilling the unique needs of each patient and improving patient outcomes and QOL.

Longitudinal studies are needed to determine the relationship between fatigue, pruritus, TD, TA, and QOL. They can help researchers understand the underlying mechanisms and potential causal relationships between these factors, as well as the bidirectional relationship between TA and complication severity. Further research is required to investigate the role of social support in TA and QOL, including the influence of family, friends, healthcare providers, and support groups. Understanding how social support affects TA and QOL can guide the development of interventions to enhance social support networks (Supplementary Information).

### Supplementary Information

Supplementary Information.

## Data Availability

The data that support the findings of this study are available from the corresponding author upon reasonable request.
